# Searching and Intertwining: Climbing Plants and GrowBots

**DOI:** 10.3389/frobt.2020.00118

**Published:** 2020-08-25

**Authors:** James Gallentine, Michael B. Wooten, Marc Thielen, Ian D. Walker, Thomas Speck, Karl Niklas

**Affiliations:** ^1^Department of Electrical and Computer Engineering, Clemson University, Clemson, SC, United States; ^2^Plant Biomechanics Group and Botanic Garden, University of Freiburg, Freiburg im Breisgau, Germany; ^3^FMF, Freiburg Materials Research Center, University of Freiburg, Freiburg im Breisgau, Germany; ^4^FIT, Freiburg Center for Interactive Materials and Bioinspired Technologies (FIT), Freiburg im Breisgau, Germany; ^5^Cluster of Excellence livMatS @ FIT – Freiburg Center for Interactive Materials and Bioinspired Technologies, University of Freiburg, Freiburg im Breisgau, Germany; ^6^School of Integrative Plant Science, Cornell University, Ithaca, NY, United States

**Keywords:** lianas, tendrils, intertwining, vines, robot, continuum, stability, grasping

## Abstract

Applications in remote inspection and medicine have motivated the recent development of innovative thin, flexible-backboned robots. However, such robots often experience difficulties in maintaining their intended posture under gravitational and other external loadings. Thin-stemmed climbing plants face many of the same problems. One highly effective solution adopted by such plants features the use of tendrils and tendril-like structures, or the intertwining of several individual stems to form braid-like structures. In this paper, we present new plant-inspired robotic tendril-bearing and intertwining stem hardware and corresponding novel attachment strategies for thin continuum robots. These contributions to robotics are motivated by new insights into plant tendril and intertwining mechanics and behavior. The practical applications of the resulting GrowBots is discussed in the context of space exploration and mining operations.

## Introduction

Traditionally, the structures of robotic appendages (arms, legs, fingers) have been based on interconnected rigid links, with the shape of the structures variable at only a small, finite number of locations (joints) (Siciliano and Khatib, 2016). The rigidity of the links and the ability to directly control the joint angles have enabled accuracy and repeatability that has made robots highly successful in numerous applications, notably in assembly operations within highly structured factory environments.

In recent years, however, the need to navigate within narrow, sensitive, and congested environments has motivated the development of a new class of “tongue, trunk, and tentacle” robots, collectively termed continuum robots (Walker, 2013a). Continuum robots feature continuous, compliant backbones that can change shape (bend and often extend/contract) at all locations along the structure. This feature allows them to adapt to and penetrate cluttered and tight spaces. However, regulating the shape of these robots can be challenging, given that only a finite number of actuators can be applied to control the (theoretically infinite) degrees of freedom present in the backbones.

The challenge of shape regulation is magnified in long, thin variants of continuum robots (Walker, 2013b). Although necessary to enable their envisioned applications [for example inspection within and behind equipment racks on the International Space Station (Wooten et al., 2018) and numerous medical procedures (Burgner-Kars et al., 2015)] the thin profile (lengths of a meter or more, with length to diameter ratio of 100 or more) of these structures renders them highly susceptible to undesired and uncontrollable shape changes as a consequence of external loading (gravity, air currents, environmental contact, etc.).

Many plants experience similar challenges. To meet them, plants have evolved a variety of structures and growth strategies. In particular, numerous climbing plants grow and deploy tendrils or tendril-like structures on their very thin stems, and use these organs to reach out to and stabilize stems via connection with structures in their surrounding environments (Putz and Mooney, 1992; Bohn et al., 2015; Schnitzer et al., 2015), or they intertwine individual stems to form braid-like structures to bridge gaps between supports. This “existence of proof” in the natural world provides many insights into potential innovative robotic solutions to similar problems. Of particular relevancy to robotic applications is the fact that stems bearing tendrils and tendril-like structures and stems with the ability to intertwine have evolved multiple times in phylogenetically unrelated plant lineages, which provides circumstantial evidence for convergent adaptive evolution by means of natural selection. This convergence in the form and function of different plant grasping organs (modified stems, leaves, and even roots) manifests different anatomical and morphological solutions for constructing thin continuum robots.

Vascular plants evolved ~350 million years ago and have been subjected to intense natural selection for this period of time (Niklas, 2016). Consequently, extinct as well as living species can be viewed as evolutionary experiments that have either failed or that have passed the test of selection. Because a primary requirement for survival on land is the ability to cope with internal and external mechanical forces (i.e., self-loading and wind-induced drag forces), the shape, size, internal structure, and behavior of plants provide opportunities to transfer organic mechanical strategies to the construction of engineered artifacts, such as robots.

An important “proof of (evolutionary) concept” is convergence—when different unrelated organisms solve the same problem using similar methods. For example, plant organs such as tendrils, stems, and leaves have evolved independently in many different land plant lineages (e.g., ferns, lycophytes, eudicots, and monocots) (Niklas, 2016). Yet, in each case these organs are made of hierarchically structured composite materials (different cell- and tissue-types) that have strain incompatibilities owing to their different elastic moduli and physical anisotropy. An emergent feature of this mode of construction is the differential storage of strain energy when organs bend or twist, either as a result of self-loading or the application of external loads (wind-induced pressure forces). The stored strain energy can be used to restore the original postures of stems and tendrils when bending forces are removed. One example of this mechanical strategy is seen in hollow stems that are subdivided into smaller cylindrical compartments by transverse diaphragms at nodes, which act as spring-like joints that store strain energy when caused to buckle under bending or twisting forces. Data from resonance frequency tests have been used to calculate spring constants for stem segments and have been shown to agree with those predicted by theory provided that nodes act as spring-like joints. When diaphragms are punctured, nodal spring constants are reduced by as much as 35%.

In this paper we introduce, for the first time, continuum robot hardware (modeled after plants with very thin stems bearing tendrils) specifically equipped with searcher stem hardware designed for environmental contact. The tendril hardware is based on the activation of pre-coiled Shape Memory Alloy (SMA) materials. We demonstrate herein that such structures, when deployed as robotic stems bearing tendrils, can effectively stabilize thin continuum robots via anchoring them to themselves (braiding) and their surroundings (stabilizing). The first development of plant-inspired SMA-based robotic searcher tendril stems, focused toward climbing and grasping robots, was reported in Vidoni et al. (2013, 2015). The work in this paper is the first time, to the best of our knowledge, that continuum robotic stems have been integrated and deployed with attached searcher stems as braiding/stabilizing elements. In this way, this paper offers a completely new approach to physical robot-environment interaction.

Section Thin Continuum Robots and the Need for Environmental Attachment summarizes the state of the art in thin continuum robotics. The discussion is intended to illustrate the critical need for new ways to anchor and stabilize robots. Related structures and strategies evolved and adopted by thin-stemmed plants are discussed in section Tendrils and Intertwining Searchers in Climbing Plants. Motivated in large part by the insight gained from this understanding of plant morphology and behavior, the new robot tendril hardware, and novel strategies for novel robotic operations using it, are presented in section Innovative Attachment with Novel Robot Tendrils. Discussion and conclusions follow.

## Thin Continuum Robots and the Need for Environmental Attachment

Inspired by the success of thin continuum robots in medical procedures (Burgner-Kars et al., 2015), and motivated by the need for remote inspection in, for example, space (Mehling et al., 2006; Wooten and Walker, 2015) and aeronautical applications (Dong et al., 2017), researchers have explored the creation of long (over a meter in length), thin (length to diameter ratios of 100 or larger) continuum robots (Mehling et al., 2006; Mazzolai et al., 2014; Tonapi et al., 2014; Wooten and Walker, 2015; Dong et al., 2017; Greer et al., 2018) that are the mechanical analogs of ultra-thin stems. When viewed as thin stem-like cantilevered beams, these efforts have been further stimulated by recent interest in creating plant-inspired robots (Sadeghi et al., 2013; Mazzolai et al., 2014; Del Dottore et al., 2018; Putzu et al., 2018). For example, when operating thin continuum robots in free space, researchers have been inspired by and therefore adopting models of the circumnutation movements observed in plants, i.e., an oscillatory rotational motion of the growing tips of plant stems that first drew the attention of Charles Darwin (Bastien and Meroz, 2016; Del Dottore et al., 2016; Wooten and Walker, 2018).

However, significant problems arise when external loadings are considered. Continuum robots, by nature of their construction and intended function, are compliant all along their length. Consequently, long and thin continuum robots typically struggle with bearing external loads, and often even with compensating for their own weight. Such thin continuum robot designs result in an effective exploration device, but as the backbone sections become longer and more numerous, the mass of the structure greatly reduces the load-bearing capacity of the robot overall. Multiple sections, especially when using spring-loaded sections, introduce coupling between these sections, i.e., as a tendon for the tip section is pulled all sections proximal to it are also affected. Additionally, with the actuation being located at the base of the structure (necessary to produce the thin profile), when actuating unconstrained thin continuum structures, the bending energy is distributed throughout the backbone, making it difficult to achieve fine control of the distal end, or tip (the equivalent of the growing apical meristem of a real plant stem).

**Figure 1:**
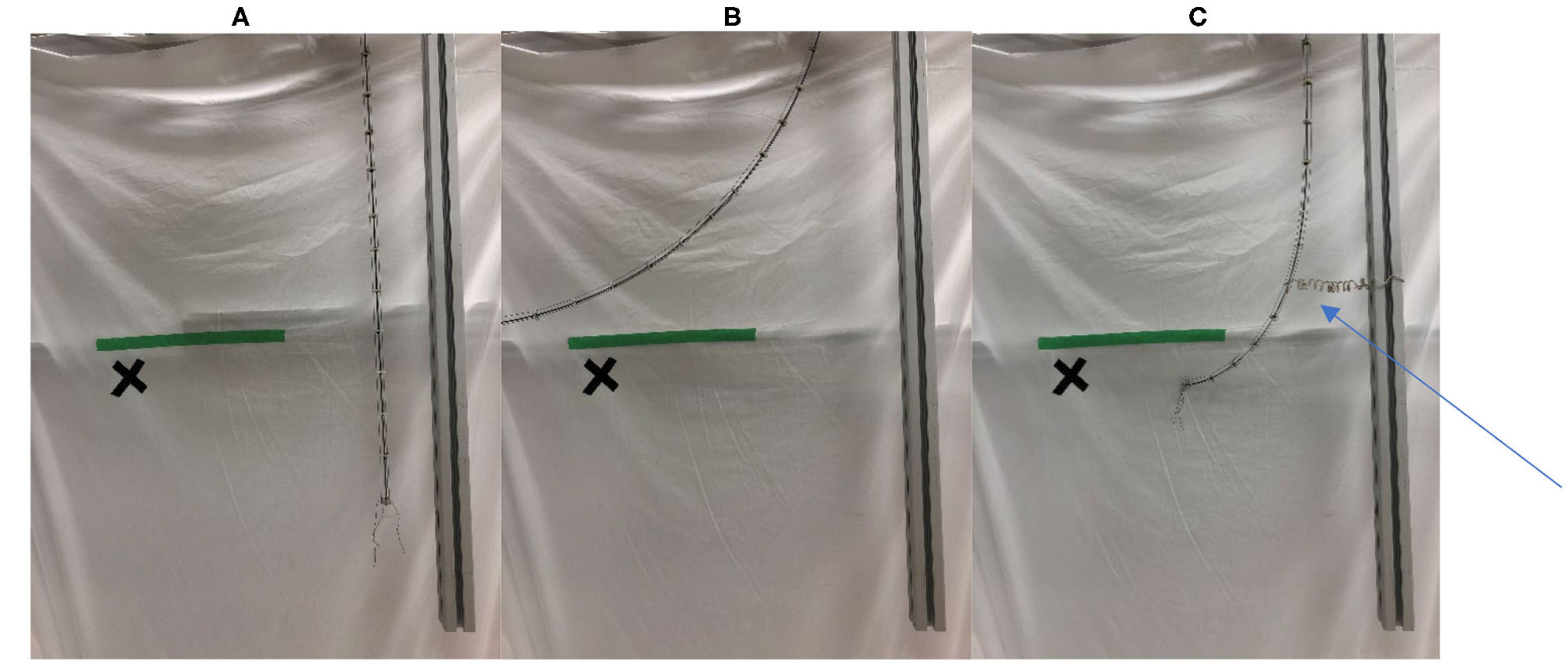
**(A)** Robot “stem” (right), fixed vertical environmental structure (far right), target location for tip camera to view marked with “x.” **(B)** Bending of unconstrained stem. Bending is along entire backbone, tip and camera pass target (to its left). **(C)** Stem with robotic “tendril” attaching it (at arrow) to environmental structure. Searcher contact stabilizes stem-tendril, allowing greater bending at the tip, such that the tip camera is able to “see” the target. The green line represents a virtual obstacle.

As an example of these issues, Figure 1 shows a tendon-actuated thin continuum design that is the mechanical analog of a very thin plant stem (the physical details of which are given in section Novel Robotics Attachment Strategies Exploiting the New Tendril Hardware), mounted vertically pointed downwards. In Figure 1A, the robot stem is to the right side, with a fixed, rigid environmental feature (a metal beam) to its right. Figure 1B shows bending of the unconstrained robot. Notice that the entire backbone bends, restricting the motion to low curvature bends, so that the robot tip is physically incapable of accessing the marked cross target point in the environment. For applications in inspection, it may be necessary to view behind a feature such as an equipment rack without contacting the rack, risking damage to either it or the robot. Figure 1C demonstrates how an attachment point in the environment constrains the motion of the continuum stem, allowing a higher curvature bend closer to the distal end of the robot. This bend allows the tip of the “stem” to view the cross target without contacting an obstacle (green line). Additionally, although an unconstrained robotic continuum “stem” can only bend with fixed curvature, environmental supports allow for more compound curvatures without the addition of more actuated segments.

An initial investigation of the above problems concluded that even a primitive means of environmental attachment (passive hooks inspired by the prickles on rose stems and tendrils on grape stems) could be used to either reduce or, in some cases, negate the above issues (Wooten and Walker, 2015, 2018). However, it proved non-trivial to attach artificial hooks onto environmental features, and a more active means to connect to the environment was sought. Figure 1C shows the novel robotic “searcher stem,” introduced in this paper, attached by a tendril, and connecting the entire assembly to its environment, allowing the tip to access (view) the marked environmental point. Note that given the environmental anchoring provided by the tendril, the searcher “stem” is stabilized, with bending distal to the anchor point largely decoupled from the proximal part of the backbone, enabling greater bending of the tip. In creating the enabling robot “searcher” stem (introduced herein in section Innovative Attachment with Novel Robot Tendrils), inspiration has been taken from climbing plants and vines, exploiting similar characteristics in their structures and their adaptive means of active environmental attachment, as discussed in the following section.

## Tendrils and Intertwining Searchers in Climbing Plants

### Tendrils, Searchers, and Intertwining

**Figure 2:**
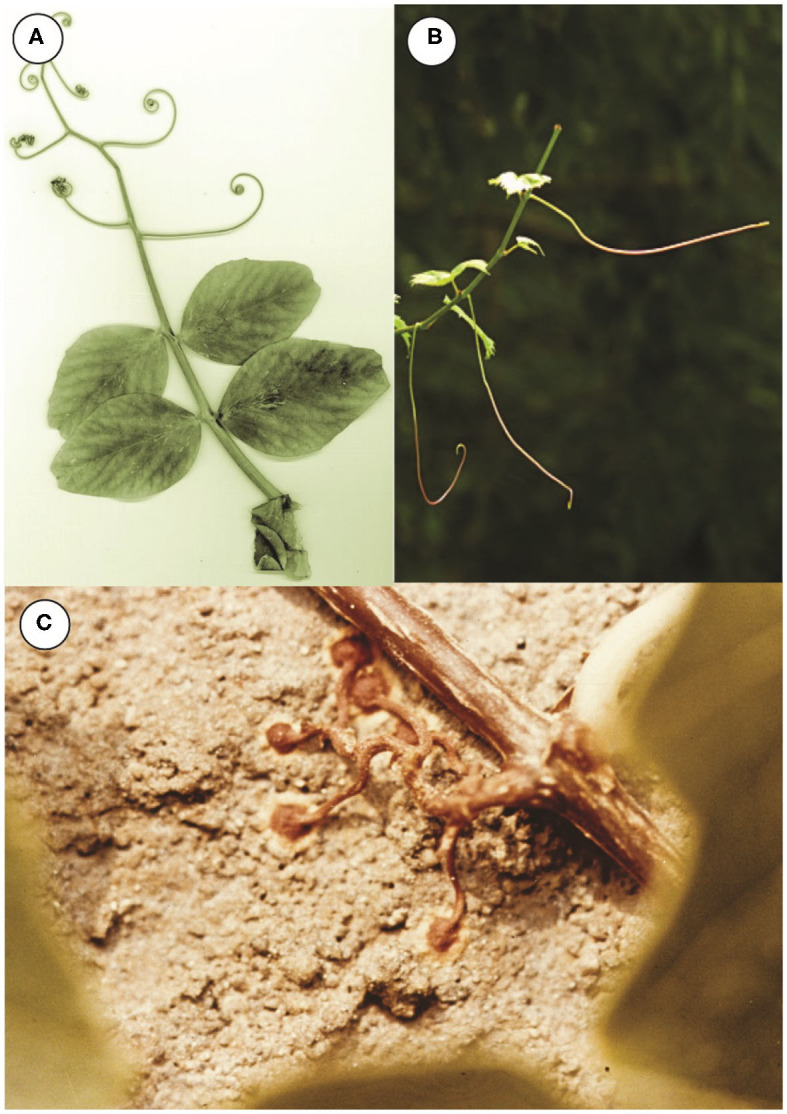
Examples of clasping plant organs. **(A)** Terminal tendrils of a pea leaf (*Pisum*). **(B)** Axillary tendril-like stems of grape (*Vitus*). **(C)** Adventitious roots of ivy (*Parthenocissus*).

Different groups of plants have adaptively converged to produce tendrils and tendril-like organs on their slender stems derived from different plant organs (i.e., leaves, stems, and even roots) (Putz and Mooney, 1992; Niklas, 2016). Modified leaves and stems are the most frequently used to achieve lateral anchorage. Examples of leaves that have been wholly or in part transformed into tendrils are seen in the common garden pea (*Pisum sativum*), which employs several pairs of leaflets of its compound leaves to develop into cylindrical tapered tendrils (Figure 2), the leaves of the Yellow Vetch (*Lathyrus aphaca*), which develop into single tendrils, and the petioles of Potato Vine (*Solanum jasminoides*) and Nasturtium (*Tropaeolum*) which develop into tendrils. In contrast, the tendrils of the common Fox grape (*Vitus*) are stems; there is either a tendril or an inflorescence opposite each leaf (see Figure 2B).

**Figure 3:**
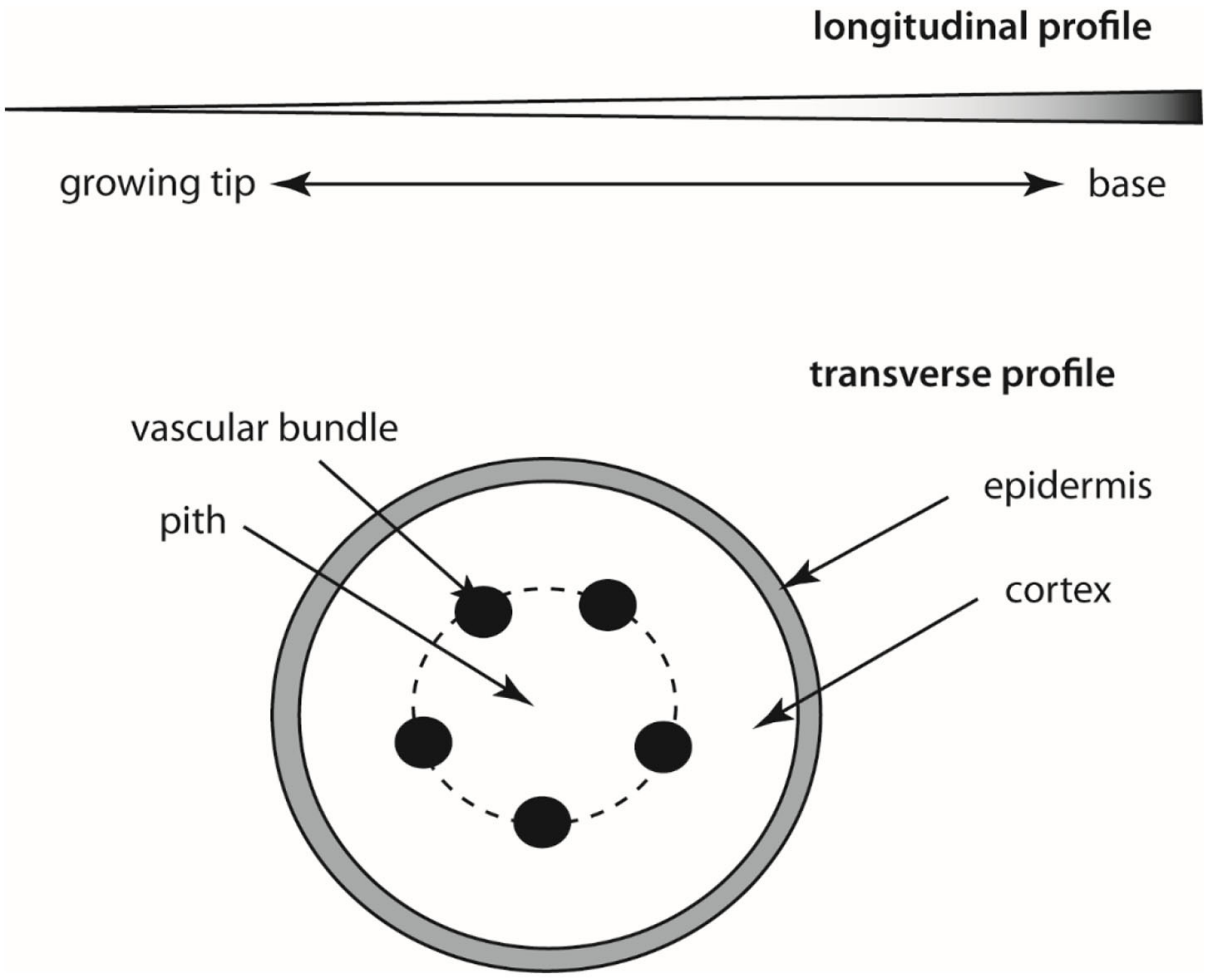
Schematic of longitudinal tapered profile **(top)** and a transverse section **(bottom)** through a typical tendril-like stem. The shading in the longitudinal section denotes increasing stiffening toward the base of the stem as a consequence of an increase in cross sectional area and lignification of tissues.

Regardless of the organ-type used to construct a tendril or tendril-like structure, commonalities in form (morphology), anatomy, and behavior are observed across phylogenetically diverse species that may instruct the engineering and construction of slender robotics. Among these commonalities are (1) tapering in girth along the length of the structure, (2) an increase in stiffness toward the base of the structure, (3) the appearance of an incompressible “core” tissue (called the pith) around which elastic rods (vascular bundles) are distributed symmetrically which are in turn compressed by a sheath of incompressible tissue (called the cortex), that is (4) ensheathed by an elastic tissue (the epidermis) (Figure 3). Circumnutation, a phenomenon explored by Charles Darwin, is also typical (i.e., the successive bending in different directions of the growing tip of the stems and tendrils of many plants, especially seen in climbing plants).

Regardless of their organographic origins, tendrils serve to find a support onto which the plant can attach and grow. This requires a mechanism to “search” the local three-dimensional space for a suitable substrate, typically with a lightweight extension sufficiently strong to support its own weight (i.e., the extension being a long antenna-like, stiff yet flexible structure). Observations of numerous climbing plant species reveal that “searcher” shoots often intertwine and provide mutual support within braided structures (Rowe and Speck, 2015). This modular construction offers mechanical advantages, such as enabling conjoined searchers to bridge larger gaps between supports than individual searcher stems could not do, and allowing individual searchers to separate from a braid, change their direction of growth, and locate different support members. Here, we investigate how such intertwined searchers can serve as role models for plant-inspired GrowBots capable of exploring three-dimensional space in which support members are randomly located.

### Morphometric Characterization and Efficiency of Intertwining Shoots

**Figure 4:**
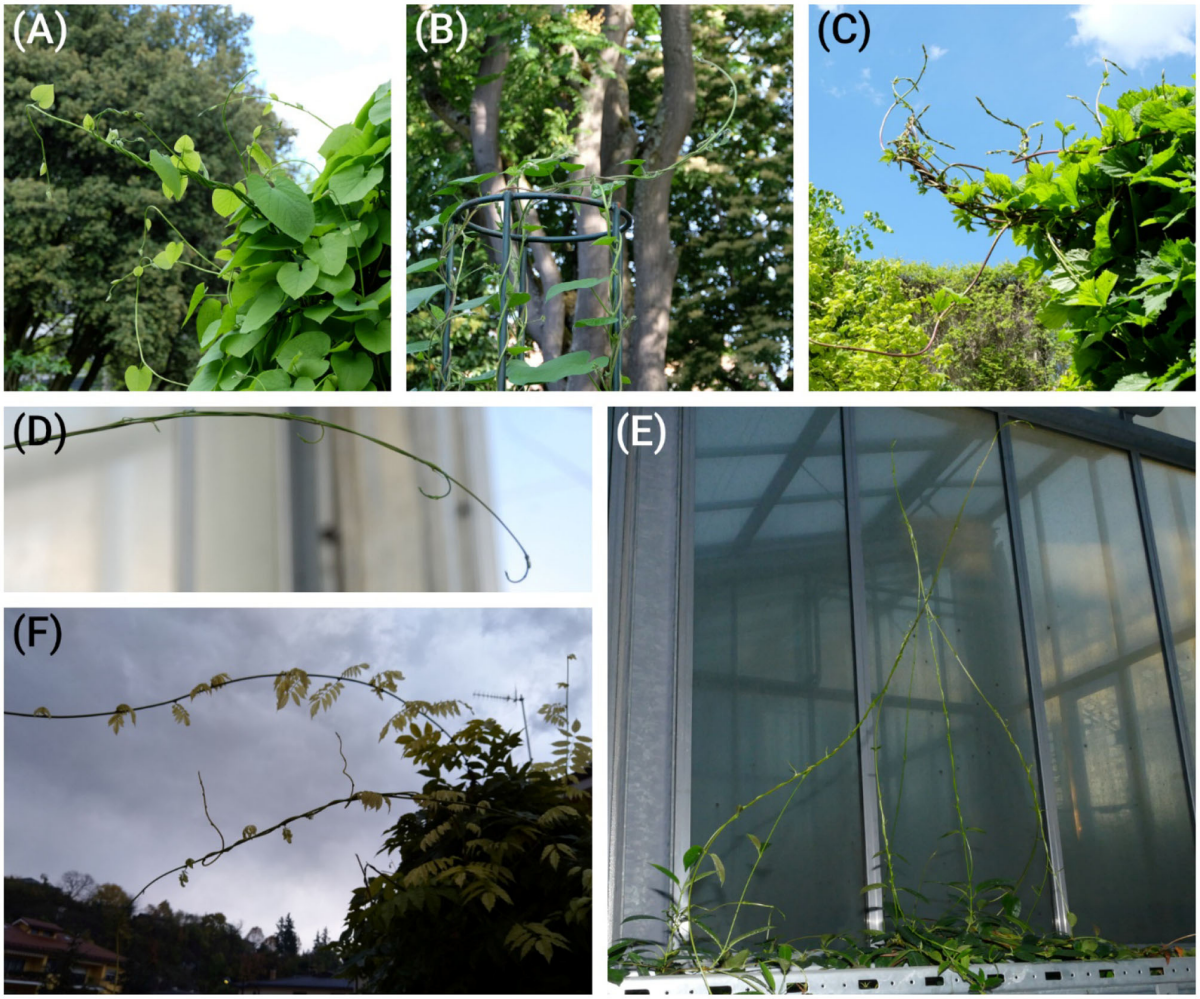
Plants with searcher stems that are intertwining and providing mutual support. *Aristolochia macrophylla* **(A)**, *Ipomoea tricolor* **(B)**, *Humulus lupulus* **(C)**, *Dipladenia* sp. **(D–E)**, and *Wisteria* sp. **(F)**.

**Figure 5:**
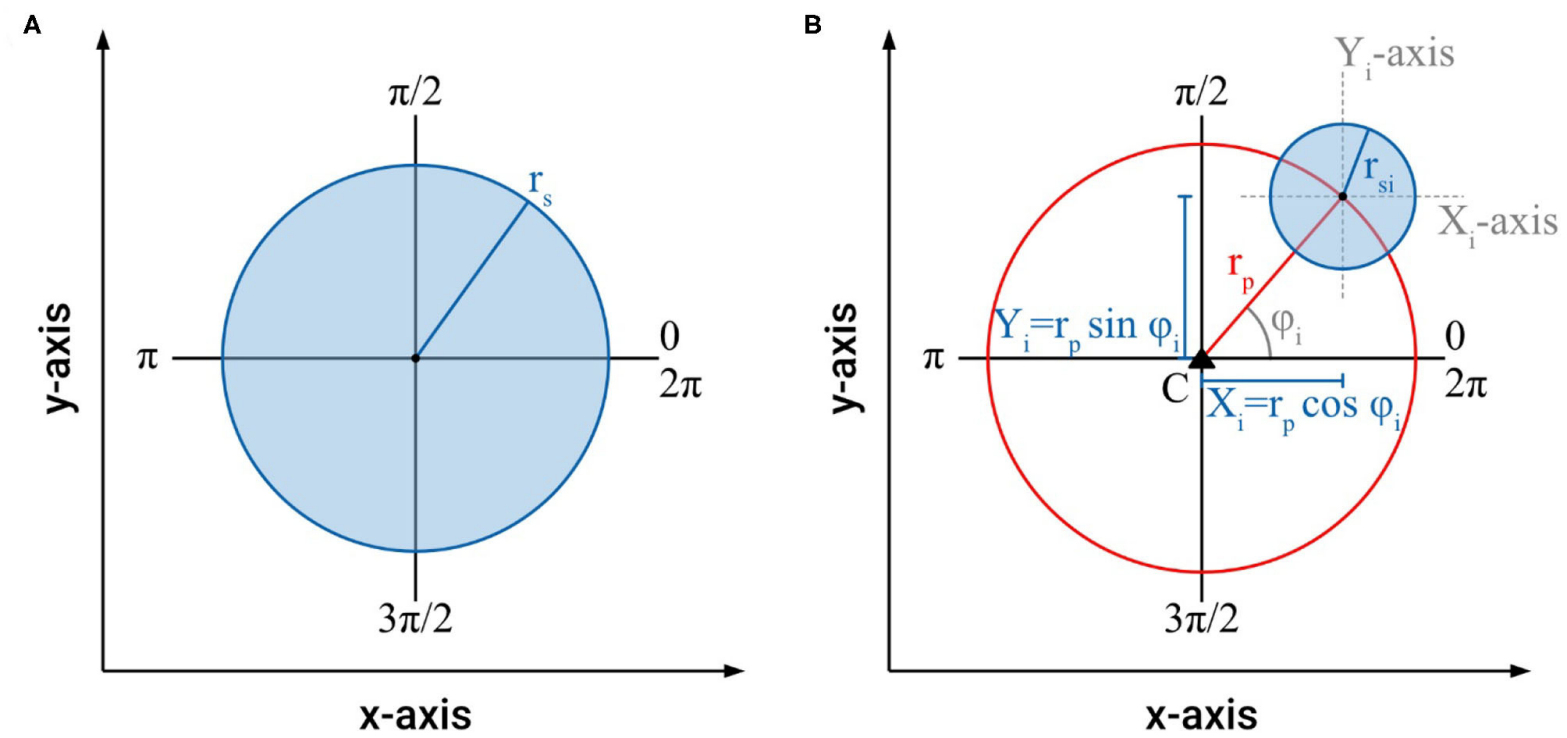
**(A)** Schematic of the cross-section of a single stem with radius *r*_*s*_ with the parameters used to calculate cross-sectional area and axial second moment of area, **(B)** schematic of the cross-section of an individual stem with radius *r*_*si*_ arranged at a distance *r*_*p*_ from the centroid *C* of a braided structure with the parameters used to calculate cross-sectional area and axial second moment of area.

Our studies show that the intertwining of searcher stems is a common phenomenon in climbing plants that increases the distance they can bridge for attaching to new host structures. Often, such “braided” structures consist of two to four intertwined searcher stems with a decreasing number of stems from base to growing tip (Figure 4). In these intertwined structures, regions with no or only very loose contact alternate with regions where all stems touch each other and form a dense cluster of stems. These regularly occurring regions of close contact ensure the coherence of the intertwined structure. In order to understand and to quantify the influence the different regions of the intertwined searcher structure (with no, distant, or close contact) have on its bending stiffness, it is helpful to characterize the morphometry of these regions. For this purpose, the axial second moments of area (= moments of inertia) for various arrangements of searcher twigs differing in number and distance from each other were calculated in a simplified approach (Figure 5). For a single shoot, a cylindrical shape with radius *r*_*s*_ is assumed (Figure 5A). Denoting the cross-sectional as *A* = π · *r*_*s*_^2^, the axial second moment of area *I*_*ax*_ is given by the equation

$$\begin{array}{l} I_{ax}=I_{x}=I_{y}=0.25\cdot \pi \cdot r_{s}{\;}^{4} \end{array}$$

In order to assess the arrangement of searching shoots in an intertwined braid, the axial second moments of area of the individual searcher stems used to construct a braid can be calculated using the Huygens–Steiner theorem, and summed for the entire system taking advantage of the additivity of axial second moments of area (Rowe and Speck, 2015; Spura, 2019). For each individual searcher stem with the radius *r*_*si*_ and cross-sectional area *A*_*i*_ = π · *r*_*si*_^2^ arranged at a distance *r*_*p*_ from the centroid *C* of the braid structure, the axial second moments of area *I*_*x*_ and *I*_*y*_ are calculated as Figure 5B:

$$\begin{array}{l} I_{x}=I_{Xi}+Y_{i}^{2}\cdot A_{i}\;\text{with }Y_{i}=r_{p}sin\varphi_{i}\;\text{and }I_{Xi}=0.25\cdot \pi \cdot r_{si}{\;}^{4}\\ I_{y}=I_{⋎i}+X_{i}^{2}\cdot A_{i}\;\text{with }X_{i}=r_{p}cos\varphi_{i}\;\text{and }I_{⋎i}=0.25\cdot \pi \cdot r_{si}{\;}^{4} \end{array}$$

In many aspects, plant searcher braids can be compared to cables or ropes, except that they typically do not have a core around which individual “wires” are wrapped (Evans et al., 2005; Costello, 2012). Based on observations of real intertwined searcher stems, the following boundary conditions were assumed to allow for an approximated calculation of the axial second moments of area of a structure consisting of *n* intertwined shoots: (1) All searcher stems are cylindrical with circular cross-sections, (2) the cross-sections of individual stems are the same for all stems and remain constant over the length of an intertwined structure, i.e., the stems have no taper, and cross-sectional area is given as *A* = *n* · π · *r*_*s*_^2^ (to simplify comparability, radii are normalized to *r*_*si*_ = *r*_*s*_ = 1), (3) the centers of gravity of the *n* intertwined stems (*cg*_1_, *cg*_2_, *cg*_3_, *cg*_4_) are at the same constant distance from the centroid of the intertwined structure *C*, i.e., they are arranged on a circle with radius *r*_*p*_, and (4) intertwined stems do not overlap and are symmetrically arranged at angles φ_*i*_ = 2π · *i/n*, with *i* = 1,2,3,4,…*n*, and (5) a searcher braid is treated as a unit in which individual stems are fixed with respect to each other.

**Figure 6:**
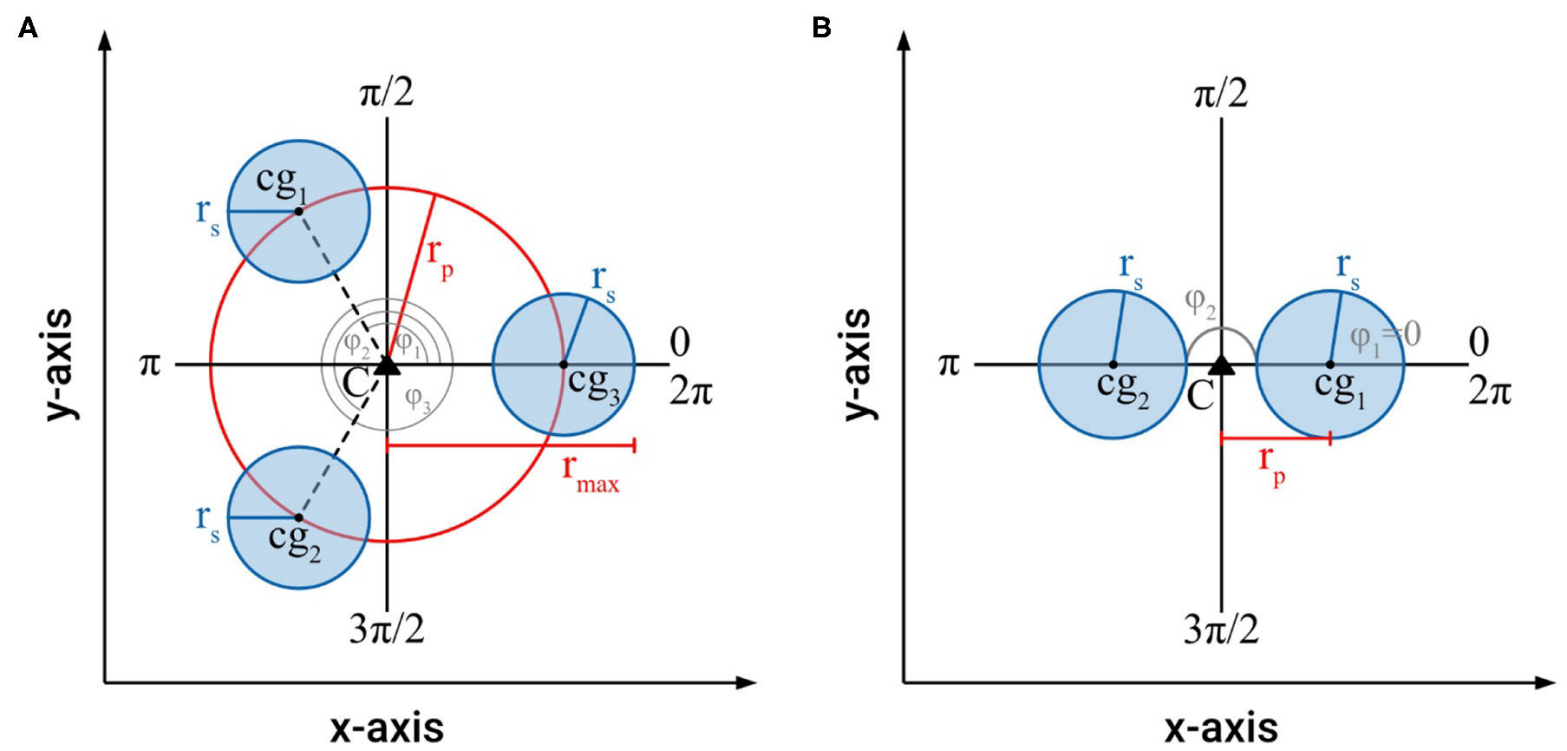
**(A)** Schematic of the cross-section of an intertwined, braided structure with a 3-fold symmetry (*n* = 3), which fulfills the boundary conditions (1)–(5), showing the parameters used to calculate cross-sectional area and axial second moment of area. **(B)** Schematic of the cross-section of an intertwined, braided structure with a 2-fold symmetry (*n* = 2), which fulfills the boundary conditions (1)–(5), showing the parameters used to calculate cross-sectional area and axial second moment of area.

For an intertwined braided structure with a 3-fold or higher symmetry, i.e., three or more individual shoots (*n* ≥ 3), which fulfills the boundary conditions (1)–(5), it had been proven that, for all reference axes through the centroid of the braided structure, the axial second moments of area are constant (Figure 6A) (Burton, 1979; Speck et al., 1990), i.e., *I*_*max*_ = *I*_*min*_ = *I*_*ax*_ = 0.5 · π · *n* · (0.5 · *r*_*s*_^4^ + *r*_*s*_^2^ · *r*_*p*_^2^). This invariance does not hold for a braided structure with two individual shoots (i.e., 2-fold symmetry with *n* = 2), for which *I*_*max*_ and *I*_*min*_ differ (Figure 6B) as follows

$$\begin{array}{ll} I_{max} & =I_{y}=2{\cdot (I_{⋎i}+X_{i}}^{2}\cdot A_{i})\;\text{with }X_{i}=r_{p}cos\varphi_{i}\;\text{and}\\ I_{⋎i} & =0.25\cdot \pi \cdot r_{s}{\;}^{4}\\ I_{min} & =I_{x}=2{\cdot (I_{Xi}+Y_{i}}^{2}\cdot A_{i})\;\text{with }Y_{i}=r_{p}sin\varphi_{i}\;\text{and}\\ I_{Xi} & =0.25\cdot \pi \cdot r_{s}{\;}^{4} \end{array}$$

The maximum distance found for real intertwined searcher stems is typically not more than 3 times the radius of an individual stem, i.e., *r*_*p,max*_ = 3 · *r*_*s*_ = 3 (for normalized *r*_*s*_ = 1).

**Figure 7:**
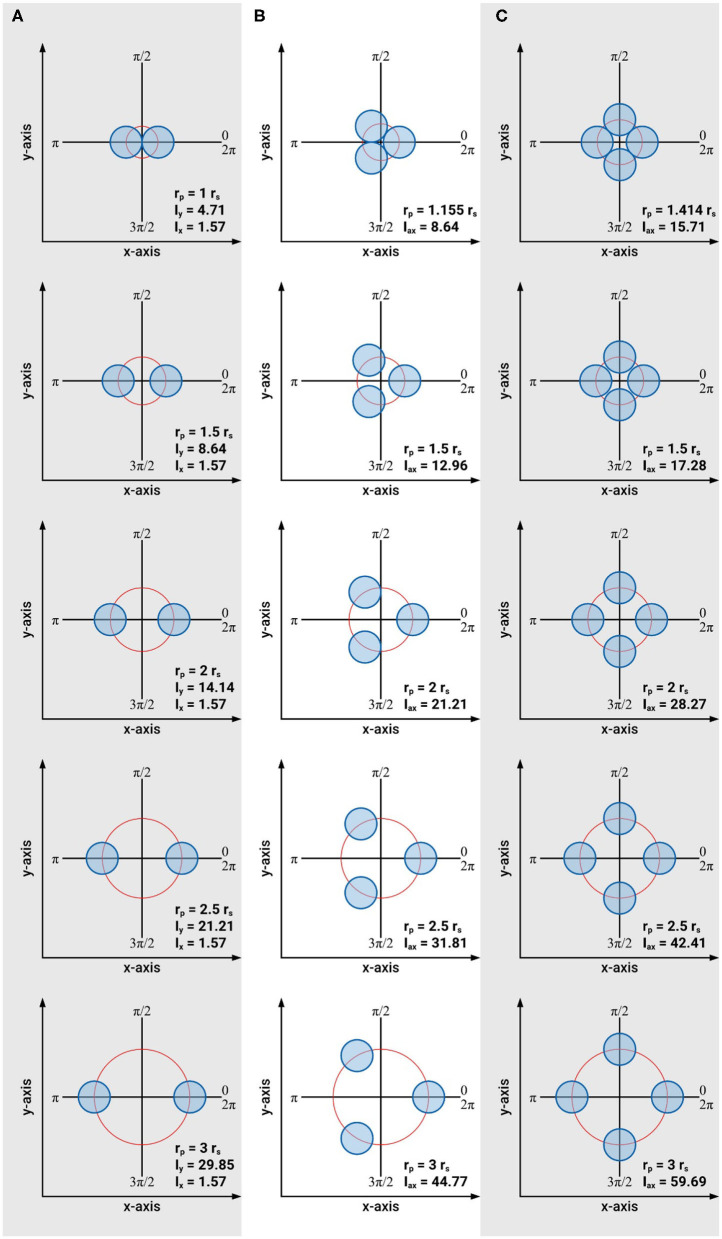
Schematic of the arrangements of intertwined braided stems consisting of one to four individual stems used to calculate axial second moments of area and braiding efficiency. **(A)** Arrangement of two intertwined stems with *r*_*s*_ = 1 = const. and *A* = 6.28 = const., **(B)** arrangement of three intertwined stems with *r*_*s*_ = 1 = const. and *A* = 9.42 = const., **(C)** arrangement of four intertwined stems with *r*_*s*_ = 1 = const. and *A* = 12.57 = const.

Based on these approximations the cross-sectional areas and axial second moments of area for an individual searcher stem with *r*_*s*_ = 1, and for two, three and four symmetrically arranged searcher stems (each with *r*_*s*_ = 1) are calculated for distances of *r*_*p*_ = *r*_*s*_, 1.5 *r*_*s*_, 2 *r*_*s*_, 2.5 *r*_*s*_, and 3 *r*_*s*_ (Figure 7). The condition *r*_*p*_ = *r*_*s*_ is only possible for two searcher stems (*n* = 2). In the case of three or four searcher stems *r*_*p*_ = *r*_*s*_ is impossible without violating boundary condition (4). By considering the arrangement of the centers of gravity on an equilateral triangle (*n* = 3) or a square (*n* = 4) it can be shown that in these cases the minimal radius of *r*_*p*_ equals *r*_*p*_ = 1.155 *r*_*s*_ (for *n* = 3) and *r*_*p*_ = 1.414 *r*_*s*_ (for *n* = 4), respectively.

Our results prove that intertwining is a very effective way to increase the axial second moment of area with minimal material investment. For two intertwined searcher stems, the maximal axial second moment of area increases by a factor of 6.34 from *I*_*max*_ = 4.71 (at *r*_*p*_ = *r*_*s*_) to *I*_*max*_ = 29.85 (at *r*_*p*_ = 3 *r*_*s*_) at constant cross-sectional area (*A* = 6.28), i.e., constant material investment. The minimal axial second moment of area, however, remains constant (*I*_*min*_ = 1.57 = const.) for *r*_*p*_ = *r*_*s*_ to *r*_*p*_ = 3 *r*_*s*_. For three intertwined searcher stems the axial second moment of area increases by a factor of 5.18 from *I*_*ax*_ = 8.64 (at *r*_*p*_ = 1.155 *r*_*s*_) to *I*_*ax*_ = 44.77 (at *r*_*p*_ = 3 *r*_*s*_), at constant cross-sectional area (*A* = 9.42), i.e., constant material investment. Finally, for four intertwined searcher stems, the axial second moment of area increases by a factor of 3.80 from *I*_*ax*_ = 15.71 (at *r*_*p*_ = 1.414 *r*_*s*_) to *I*_*ax*_ = 59.69 (at *r*_*p*_ = 3 *r*_*s*_), at constant cross-sectional area (*A* = 12.57), i.e., constant material investment. For an individual searcher stem with *r*_*s*_ = 1, typically forming the apical region of an intertwined structure, the axial second moment of area is *I*_*ax*_ = 0.785 with a cross-sectional area of *A* = 3.14.

These idealized calculations show that the axial second moment of area (a parameter that determines the contribution to flexural stiffness made by geometry, shape, and size) can be markedly increased by intertwining. Compared to an individual searcher, for the distance of individual stems to the centroid *C* of a braided structure of *r*_*p*_ = 1.5 *r*_*s*_, the axial second moment of area increases by a factor of 11.0 for two stems (*n* = 2, here *I*_*max*_ is used for comparison), a factor of 16.5 for three stems (*n* = 3), and a factor of 22.0 for four intertwined stems (*n* = 4). For the largest distance to the centroid *C* of a braided structure with individual stems of *r*_*p*_ = 3 *r*_*s*_, the axial second moment of area increases by a factor of 38.0 for two stems (*n* = 2, here *I*_*max*_ is used for comparison), a factor of 57.0 for three stems (*n* = 3), and a factor of 76.0 for four intertwined shoots (*n* = 4).

**Figure 8:**
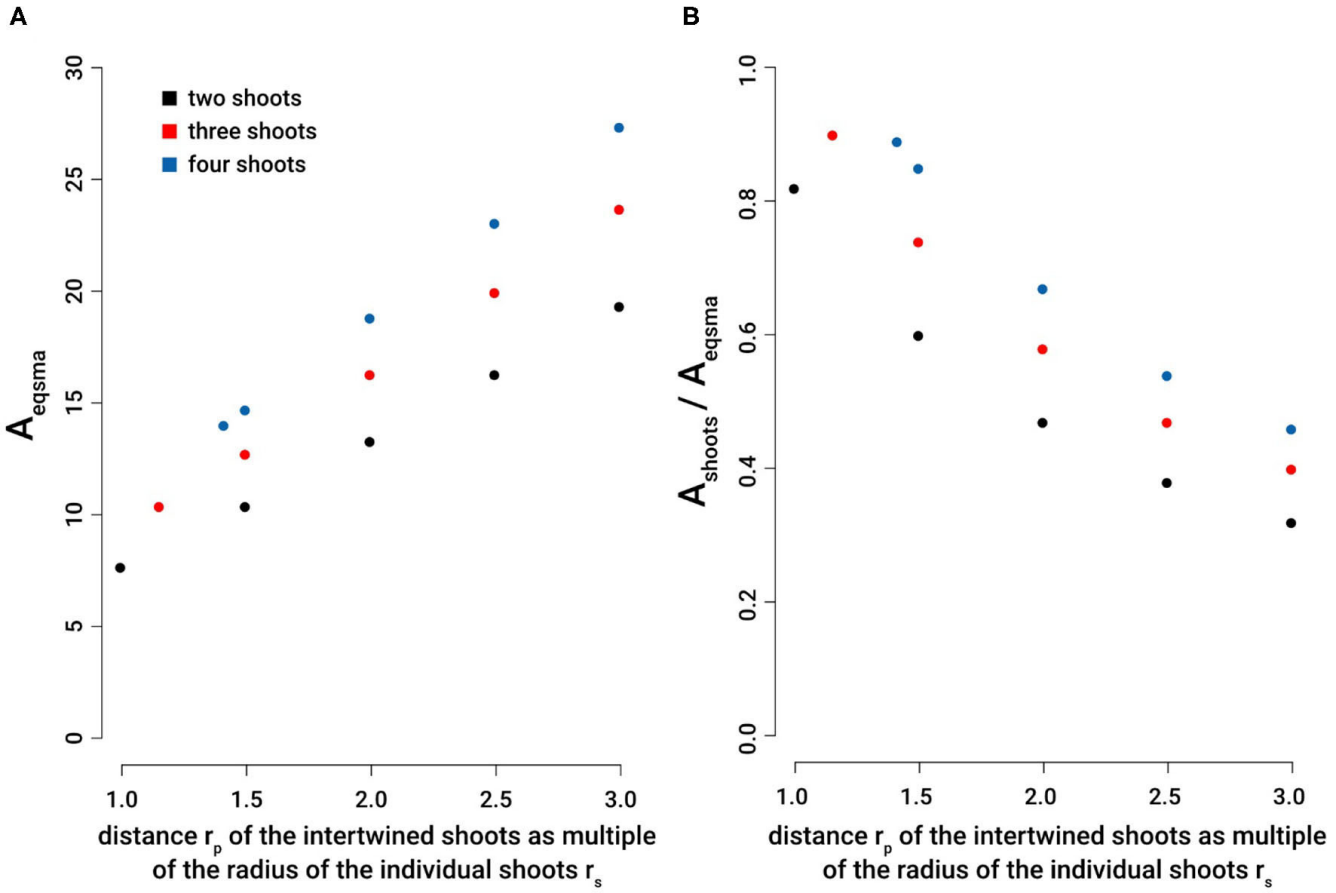
Efficiency of intertwining stems: Comparison of the cross sectional area of an individual stem required to reach the second moment of area equivalent to two, three, or four intertwined stems with various distances from the centroid of the braid structure. **(A)** A_eqsma_: cross-sectional area equal to the second moment of area of an individual shoot necessary to reach the same axial second moment of area of 2, 3, or 4 intertwined stems, each with a constant cross-sectional area of A_individualshoot_ = 3.14. **(B)** A_shoots_/A_eqsma_: ratio of cross-sectional areas comparing the cross-sectional area of 2, 3 and 4 intertwined shoots with the cross-sectional areas of equal second moment of area of an individual single shoot. The distance of intertwined stems is given by *r*_*p*_ as multiples of the radius of individual stems *r*_*s*_: *r*_*p*_ = *r*_*s*_ [or *r*_*p*_ = 1.155 *r*_*s*_ (for *n* = 3) and *r*_*p*_ = 1.414 *r*_*s*_ (for *n* = 4), respectively], *r*_*p*_ = 1.5 *r*_*s*_, *r*_*p*_ = 2 *r*_*s*_, *r*_*p*_ = 2.5 *r*_*s*_, and *r*_*p*_ = 3 *r*_*s*_.

To compare the efficiency of intertwining, it is convenient to compare the amount of material an individual single stems must invest to gain the same axial second moment of area as that of intertwined stems. If the individual stems are approximated as cylinders with no taper, a comparison of the respective cross-sectional areas of the intertwined structures at different *r*_*p*_ with the ones of an individual stem with the same axial second moments of area can be used for comparison (Figure 8). Our calculations prove that for all considered cases, the cross-sectional area of an individual stem with the same axial second moment of area is markedly higher than the one summed up for intertwined stems. This comparison shows that intertwining represents a very efficient way to increase axial second moment of area with minimized material invest. The assumptions made for these calculations reflect reality quite well in most cases. However, it should be noted that in boundary condition (2) (i.e., the assumption that all cross-sections are equal) is not always valid. Nevertheless, it represents a good first order approximation. Direct observations of real plants indicate that cross-sectional areas sometimes differ when several braids merge, or when a single searcher-shoot joins an existing braid (see Figure 4E). One aspect that significantly can influence bending stiffness is the way individual shoots interact. If they move against each other, bending stiffness may be considerably lower as compared to a situation in which they do not move against each other (cf. condition 5). In order to fully understand the form-structure-function relationships of the searcher braids it is therefore essential to determine and understand friction between individual shoots, which is subject of current research. It was assumed that fixed searcher-shoots in braids (cf. condition 5) represent an upper estimate of the flexural stiffness and was used here to highlight the maximum potential of intertwining.

To assess the mechanical bending properties of intertwined shoots of real leafy stems, three-point-bending tests were performed on segments of *Dipladenia* sp. braids using a universal testing machine (Instron 4466-10 kN, with a retrofit kit to inspect-DC standard, Hegewald & Peschke Mess- und Prüftechnik GmbH, Nossen, Germany). Stems were prevented from untwining during the handling of braid segments by loosely tying them together with yarn. The results show an increase in flexural stiffness from 8 Nmm^2^ for one stem, to 76 Nmm^2^ for two stems, to 178–533 Nmm^2^ for three intertwined stems up to 1,650 Nmm^2^ for four intertwined stems, corresponding to an increase in bending stiffness from one individual stem by the factors of 10, 22–67, and 206 to two, three, and four stems, respectively. The increases in stiffness observed for two and three intertwined stems agrees well with the calculated increases in the axial second moments of area, whereas the increase in the stiffness of four stems is markedly greater than that predicted by theory. This finding can be explained by the fact that, within the range of one, two and three stems, the elastic modulus does not change markedly, which can be expected for young, still growing searcher stems, whereas, in the case of four intertwining stems, lignification increases proximally (from the growing tip toward the base), resulting in a greater bulk tissue elastic modulus contributing to flexural stiffness. The efficiency gained beyond producing four intertwining stems disproportionally decreases with an increase in the number of stems, which may help to explain why the number of intertwining stems of real plants typically ranges between two and four and very rarely exceeds six.

## Innovative Attachment With Novel Robot Tendrils

### New Robot Stem-Tendril Hardware

Motivated by the desire to produce capabilities in robots analogous to those seen and described above in plants, we considered several approaches to developing simple robotic “searcher” stems. We ultimately developed prototype hardware searchers based on shape-memory alloy (SMA) materials, as detailed in this section.

Numerous approaches to exploiting SMA materials in robotics have been proposed in the literature (Kheirikhar et al., 2011; Coral et al., 2012; Cianchetti, 2013). In particular, the use of SMA materials as actuators and their characterization has been reported (Russell and Gorbet, 1995; Ho and Desai, 2009; She et al., 2016). SMA materials feature the property that, when heated, they return to a pre-deformed shape. Heated SMA wire will pull to its pre-trained form (herein, a spring shape) regardless of plastic deformation at lower temperatures. This physical property offers the potential for designing programmable and repeatable behavior that can mimic behaviors observed in biological searcher stems bearing tendrils or forming intertwined braid-like structures.

Numerous SMA materials have been developed previously, and, in this work, we use nickel-titanium alloy (also referred to as NiTi or Nitinol) wires. As this SMA material is heated to 46°C, it undergoes a martensite phase transformation that causes the alloy to revert into its predetermined (“pre-trained”) shape. Training of the SMA is achieved by passing current through the alloy until it heats to a temperature around 500°C. The wire is held at this temperature briefly, while setting the desired shape. After this process is completed and the wire cools to the ambient temperature, the wire may be arbitrarily plastically deformed. Upon heating, the wire will revert to the coiled shape in which it was trained.

**Figure 9:**
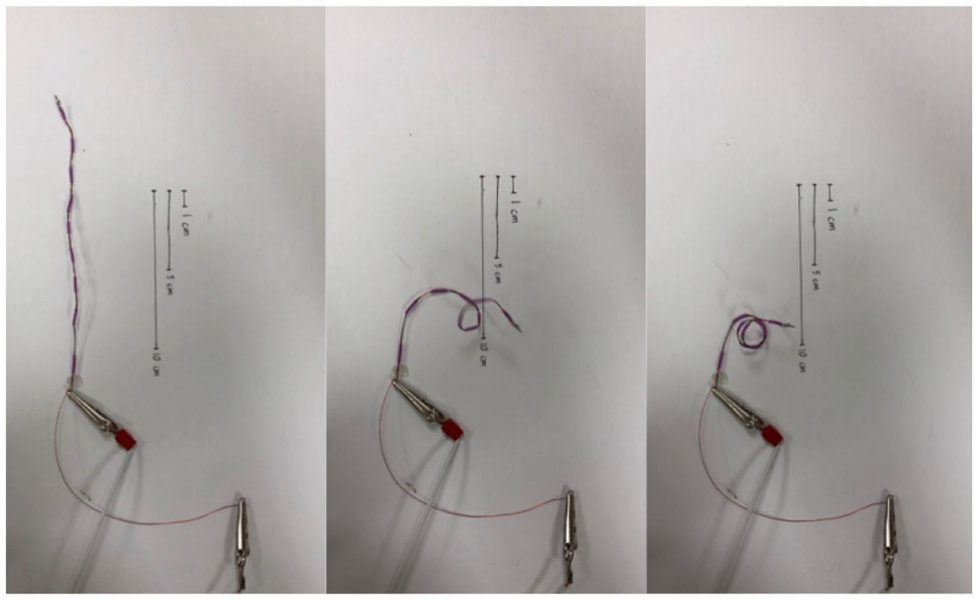
Coiling SMA robotic searcher.

Robotic searcher stems were constructed from SMA wire, being pre-trained to behave (coil into springs) similarly to the biological searchers that were their inspiration. The robotic searchers are electrically actuated. As the SMA wire conducts electricity, heat is produced which activates the searcher and causes it to move into its trained shape (Figure 9). A brief current from a 16-V, 30-watt doorbell transformer, limited by 2 amp fuses, allowed the tendril to contract fully in ~1 s.

With no current running in the searcher and the SMA at room temperature, each searcher was capable of supporting a 100-g static load hung from the actuator. Once actuated, each searcher was capable of supporting 200 g of static load.

These SMA actuators may be electrically trained into coils which mimic the shapes and behaviors of a variety of biological searchers or tendrils. This can include normal coils and shapes which mimic the reversing pitch of the robots' biological counterparts.

To mimic behaviors seen in plant searchers and tendrils, the SMA wire was trained in a tight coil. As current is passed through the searcher after it has been straightened, the SMA coil contracts, and attempts to wrap around any object that it encounters. When mounted on a GrowBot (see section Novel Robotics Attachment Strategies Exploiting the New Tendril Hardware), this coiling may be used to support the GrowBot as shown in Figure 1C, reducing the effects of external forces such as gravity and external loading on robot operation. Additionally, it can be used to form a stiffer braid-like structure on demand by intertwining several SMA wires.

Reversibility may be programmed into the SMA actuators to increase their efficacy in robotics applications, although this option was not explored in the work reported here. Preliminary results show that this could be achieved by combining a straight section of SMA with a pre-coiled elastic backbone. As the searcher is heated, the backbone would be stretched straight. Upon cooling, the elasticity of the backbone would contract the SMA into the backbone's coiled shape. This method would allow for easy construction of a variety of shapes such as the reversing pitch of the searcher's biological counterparts.

### Novel Robotics Attachment Strategies Exploiting the New Tendril Hardware

The continuum GrowBot integrated with the robotic searchers of section New Robot Stem-Tendril Hardware is a thin continuum searcher stem (sometimes referred to as a tendril in the robot literature) based on concentric tubes. Its backbone consists of one to three concentric carbon fiber tubes, with the tubes of smaller radius oriented toward the distal end. Each independently actuated section has three tendons terminating at its tip. Tendons for each section are strung through 3-D printed spacers that run along the backbone. Tendons for distal tubes are strung through proximal spacers to the base.

**Figure 10:**
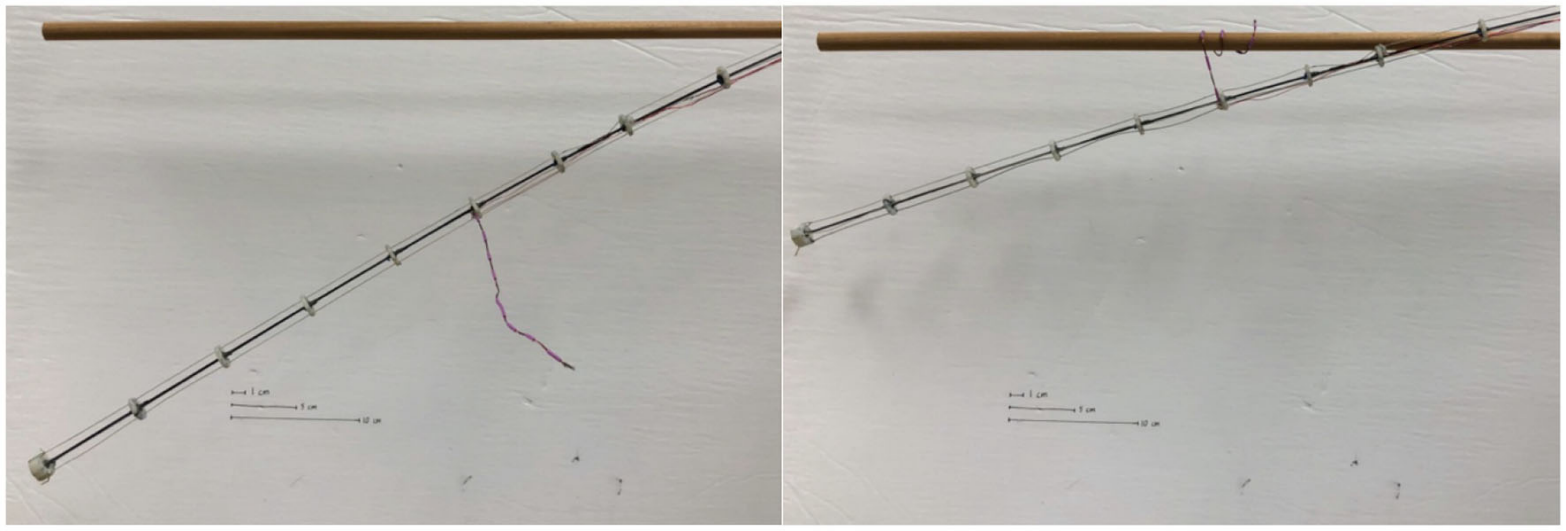
Robot tendril grasping environment with a stabilizing searcher.

**Figure 11:**
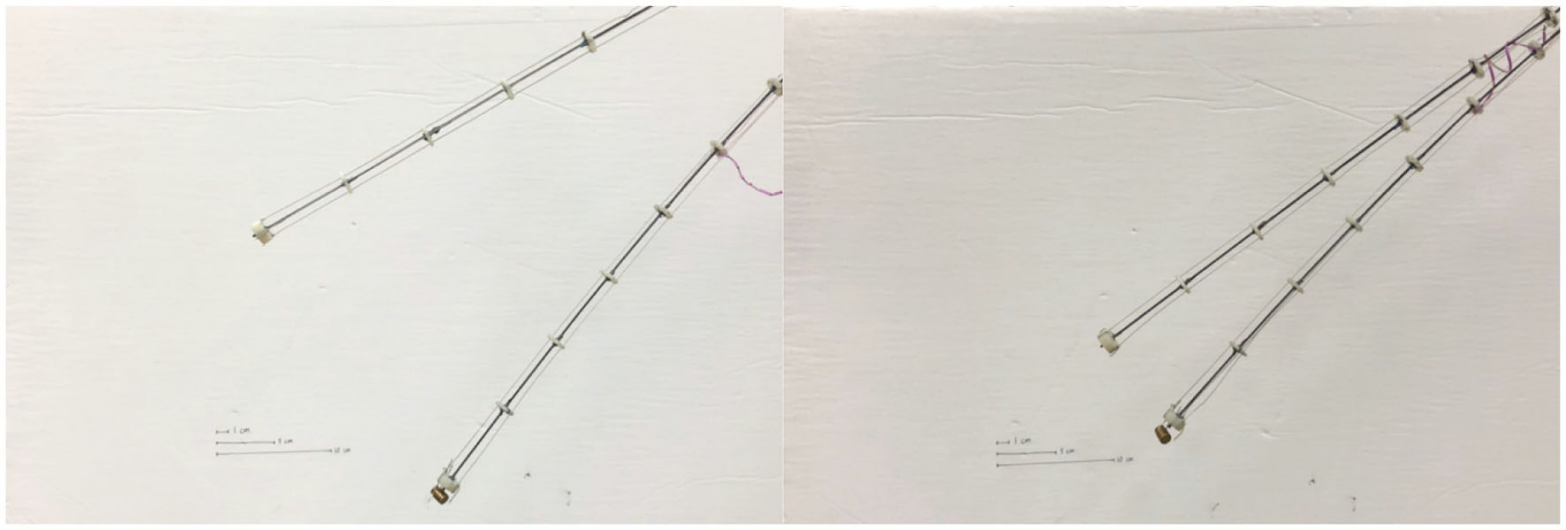
Robot tendril using SMA searcher to braid with a parallel tendril.

Robotic searchers of between 7 and 9 mm in (undeformed) length were attached to the spacers of the tendril at various points along the tendril's length and teleoperation was used to anchor the tendril to either fixed supports present in the environment (Figure 10) or to other spatially close tendrils (Figure 11). Weight at the tip of the tendril caused displacement which allowed for comparison of stiffness when the tendril is braced on its environment using stabilizing searchers.

To quantify the ability of the searchers to improve the load capacity of the system, a series of experiments was conducted, varying the mass of a tip load on the system, for unconstrained and anchored cases with both rigid environmental supports and other collocated tendrils.

**Figure 12:**
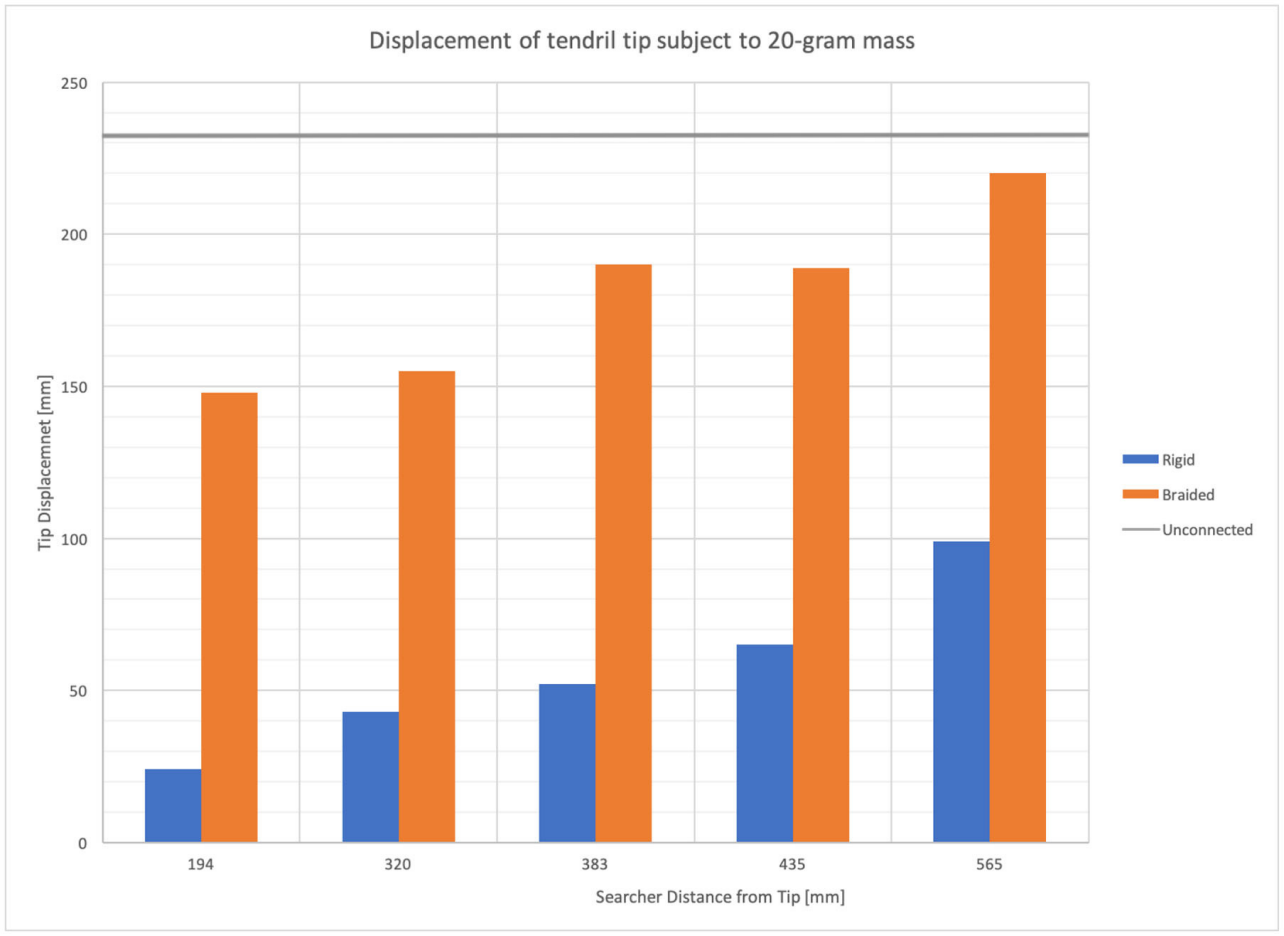
Stem-tendril's tip displacement when subjected to a 20-g mass at the tip. Comparison of displacements in cases where a searcher stem is connected at increasing distances (left to right) from the tip, to a rigid environmental object (blue bars), and when braided to a second stem-tendril (orange bars). Displacement when unconnected shown as gray horizontal line.

Figure 12 provides the displacement of the tip of a stem-tendril robot when subject to a 20-g mass at the tip of a horizontally mounted tendril as a function of the distal displacement of the searcher. The stem-tendril was stabilized using either its environment or via being braided to a second stem-tendril which was mounted parallel to the first. In these experiments, the stem-tendril experienced displacement from tip to searcher location. The searcher was initially taut, and did not experience displacement. As the searcher was moved away from the distal portion of the stem-tendril, the stem-tendril's structure was closer to that of an unsupported stem-tendril. As the searcher approached the tip, there was an increase in overall stiffness of the stem tendril. These results are consistent with the findings from braiding with plant structures reported in section Morphometric Characterization and Efficiency of Intertwining Shoots.

**Figure 13:**
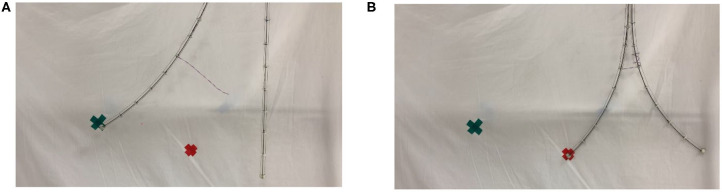
**(A)** Unconstrained left searcher reached the green (left) cross marker, but not reaching the red (right) cross marker. **(B)** Braided searcher pair, using antagonistic actuation, creates a smaller bending radius, enabling the left searcher to reach the red cross marker.

Figure 13 further illustrates the potential of braiding (intertwining) between two stem-tendril robots using the robot searchers. In Figure 13A, the left most searcher stem is actuated, and can reach the leftmost (green) marked target. However, it is too long and cannot bend sufficiently to reach the rightmost (red) marker target. However, when braided with the searcher, and working antagonistically—left stem bending to the left, right stem bending to the right, locally greater curvature is now achievable and the left searcher stem can reach the other target (Figure 13B). Note that overall the left most searcher curvature in Figure 13B is less than in Figure 13A. However, the effect of the braiding allows the creation of sections of different length, and hence different curvatures (straighter and more curved, respectively, in Figure 13B), allowing the robot to reach configurations not otherwise available.

The simple experiment illustrated in Figure 13 demonstrates the potential for searchers to provide self-stabilization in multiple intertwining structures via twining/braiding. This offers a completely new robot/robot interaction mode, in addition to the novel robot/environment interaction illustrated in Figure 9.

## Discussion and Conclusions

This paper introduced novel plant-inspired “searcher” stem-tendril robots, based on Shape Memory Alloy (SMA) materials. The robotic searchers were attached to and employed to stabilize robotic tendrils. This allows the proximal section of the robot (between the searcher and base) to be effectively isolated from the effects of outside forces. The remaining distal portion of the searcher is then able to act as a smaller unit, achieving tighter bending radii, finer positional control, and greater stability under load. Calculations based on intertwined plant searchers show that the interaction of individual and essentially independent subunits leads to an increase in efficiency in terms of weight, which in turn allows a higher reach of the “braided” structure. The independent appearance of vines and tendril-like organs among many different plant lineages (e.g., lycophytes, ferns, gymnosperms, and flowering plants) provides evidence for the adaptive nature of these structures and it provides inspiration for adopting these organic structures as models for future robotics.

## Data Availability Statement

The datasets generated for this study are available on request to the corresponding author.

## Author Contributions

JG: robot tendril design and hardware and experimental results with robots. MW: robot stem-searcher hardware and experimental results with robots. IW: development of robotic twining concept and robot design. TS and MT: theoretical and experimental studies on intertwining of searcher stems in climbing plants and contribution to ideas for transferring these structural features to novel plant-inspired GrowBots. KN: insight and understanding of structures and adaptations of plant tendrils and searchers. All authors: contributed to the first draft of the manuscript, improved further versions, and gave final approval for publication.

## Conflict of Interest

The authors declare that the research was conducted in the absence of any commercial or financial relationships that could be construed as a potential conflict of interest. The reviewer BM declared a past co-authorship with the authors IW and TS to the handling Editor.
